# A lipid/PLGA nanocomplex to reshape tumor immune microenvironment for colon cancer therapy

**DOI:** 10.1093/rb/rbae036

**Published:** 2024-03-28

**Authors:** Nan Zhang, Qiqi Sun, Junhua Li, Jing Li, Lei Tang, Quan Zhao, Yuji Pu, Gaofeng Liang, Bin He, Wenxia Gao, Jianlin Chen

**Affiliations:** Henan Academy of Sciences, Zhengzhou 450046, China; National Engineering Research Center for Biomaterials, College of Biomedical Engineering, Sichuan University, Chengdu 610064, China; National Engineering Research Center for Biomaterials, College of Biomedical Engineering, Sichuan University, Chengdu 610064, China; National Engineering Research Center for Biomaterials, College of Biomedical Engineering, Sichuan University, Chengdu 610064, China; National Engineering Research Center for Biomaterials, College of Biomedical Engineering, Sichuan University, Chengdu 610064, China; National Engineering Research Center for Biomaterials, College of Biomedical Engineering, Sichuan University, Chengdu 610064, China; National Engineering Research Center for Biomaterials, College of Biomedical Engineering, Sichuan University, Chengdu 610064, China; National Engineering Research Center for Biomaterials, College of Biomedical Engineering, Sichuan University, Chengdu 610064, China; Henan Academy of Sciences, Zhengzhou 450046, China; National Engineering Research Center for Biomaterials, College of Biomedical Engineering, Sichuan University, Chengdu 610064, China; School of Pharmacy, Chengdu University, Chengdu 610106, China; School of Laboratory Medicine, Sichuan Provincial Engineering Laboratory for Prevention and Control Technology of Veterinary Drug Residue in Animal-origin Food, Chengdu Medical College, Chengdu 610500, China

**Keywords:** lipid/PLGA nanocomplex, immune checkpoint blockade, photodynamic therapy, IDO inhibition, colon cancer

## Abstract

Immune checkpoint blockade therapy provides a new strategy for tumor treatment; however, the insufficient infiltration of cytotoxic T cells and immunosuppression in tumor microenvironment lead to unsatisfied effects. Herein, we reported a lipid/PLGA nanocomplex (RDCM) co-loaded with the photosensitizer Ce6 and the indoleamine 2,3-dioxygenase (IDO) inhibitor 1MT to improve immunotherapy of colon cancer. Arginine–glycine–aspartic acid (RGD) as the targeting moiety was conjugated on 1,2-distearoyl-snglycero-3-phosphoethanolamine lipid via polyethylene glycol (PEG), and programmed cell death-ligand 1 (PD-L1) peptide inhibitor DPPA (sequence: CPLGVRGK-GGG-d(NYSKPTDRQYHF)) was immobilized on the terminal group of PEG via matrix metalloproteinase 2 sensitive peptide linker. The Ce6 and 1MT were encapsulated in PLGA nanoparticles. The drug loaded nanoparticles were composited with RGD and DPPA modified lipid and lecithin to form lipid/PLGA nanocomplexes. When the nanocomplexes were delivered to tumor, DPPA was released by the cleavage of a matrix metalloproteinase 2-sensitive peptide linker for PD-L1 binding. RGD facilitated the cellular internalization of nanocomplexes via a_v_β_3_ integrin. Strong immunogenic cell death was induced by ^1^O_2_ generated from Ce6 irradiation under 660 nm laser. 1MT inhibited the activity of IDO and reduced the inhibition of cytotoxic T cells caused by kynurenine accumulation in the tumor microenvironment. The RDCM facilitated the maturation of dendritic cells, inhibited the activity of IDO, and markedly recruited the proportion of tumor-infiltrating cytotoxic T cells in CT26 tumor-bearing mice, triggering a robust immunological memory effect, thus effectively preventing tumor metastasis. The results indicated that the RDCM with dual IDO and PD-L1 inhibition effects is a promising platform for targeted photoimmunotherapy of colon cancer.

## Graphical abstract

**Figure:**
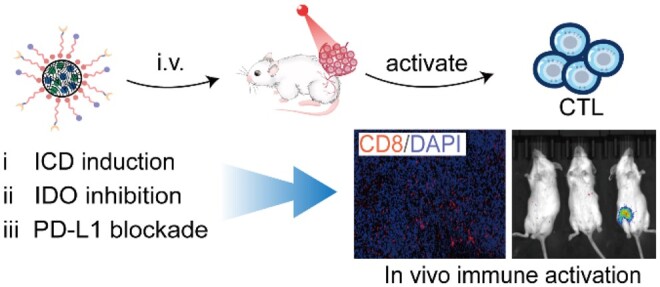

## Introduction

Immune escape is a crucial feature of tumor, in which tumor cells establish multiple mechanisms to evade host immune surveillance utilizing its heterogeneity and genetic variability, such as the expression of inhibitory receptors programmed cell death-ligand 1 (PD-L1), cytotoxic T lymphocyte-associated antigen 4 and indoleamine 2,3-dioxygenase (IDO) [1–3]. Immune checkpoint blockade (ICB) therapy activates the immune system by inhibiting immunosuppressive co-inhibitors, and has achieved remarkable efficacy in clinical applications. Nevertheless, ICB therapy could only benefit a few proportion of patients (response rate: 10–40%) [4–6]. Limited tumor infiltrating cytotoxic T lymphocytes (CTLs) and immunosuppressive tumor microenvironment (TME) are the two major challenges for ICB therapy. IDO is a rate-limiting enzyme that catalyzes tryptophan (Trp) to kynurenine (Kyn), and it is expressed in normal tissues to induce immune tolerance and prevent tissue damage [7, 8]. IDO overexpression in tumor cells can inhibit the activation of T cells and the recruitment of regulatory T cells (Tregs) through the Kyn pathway by depleting Trp, leading to tumor proliferation and immune escape [9–12]. 1-Methyl-D-tryptophan (1MT) is a competitive IDO inhibitor that has been awarded the title of orphan drug by the US Food and Drug Administration (FDA) for the treatment of stage IIb to IV melanoma. Clinical studies indicated that the antitumor effect of 1MT is weak when used alone, but its antitumor efficiency is significantly enhanced when used in combination with other therapies such as ICB therapy and photodynamic therapy (PDT) [13–16]. However, it is hindered by poor water solubility, lack of targeting, and inability to accumulate in tumor sites.

PDT damages tumor cells through reactive oxygen species (ROS) generated by photosensitizer under laser irradiation, its advantages are minimally invasive, spatiotemporal selectivity and low systemic toxicity [17, 18]. Relevant studies showed that PDT could induce immunogenic cell death (ICD) while killing tumor cells to release damage-associated molecular patterns (DAMPs) such as transfer of calreticulin (CRT) from the cytoplasm to the cell surface, secretion of high-mobility group box 1 protein (HMGB1) and adenosine triphosphate (ATP), etc. ICD promotes dendritic cells (DCs) maturation and CTLs activation, proliferation, and differentiation, and ultimately activates the immune system to produce a synergistic effect with ICB therapy [19–22].

Chlorin e6 (Ce6) is an FDA-approved photosensitizer [23–25]. The *in vivo* application of Ce6 is limited by the poor water solubility and insufficient accumulation in tumor sites. Nano-drug delivery systems are designed to prolong the circulation time of drugs *in vivo*, enable targeted delivery of multiple therapeutics to tumor sites and tumor-specific payload release [26–31]. Lipid/polymer nanocomplex is a promising nano-drug delivery system that combines the advantages of liposomes and polymer nanoparticles such as high drug loading, good biocompatibility, and easy manufacturing process [32–35].

**Figure 1.**
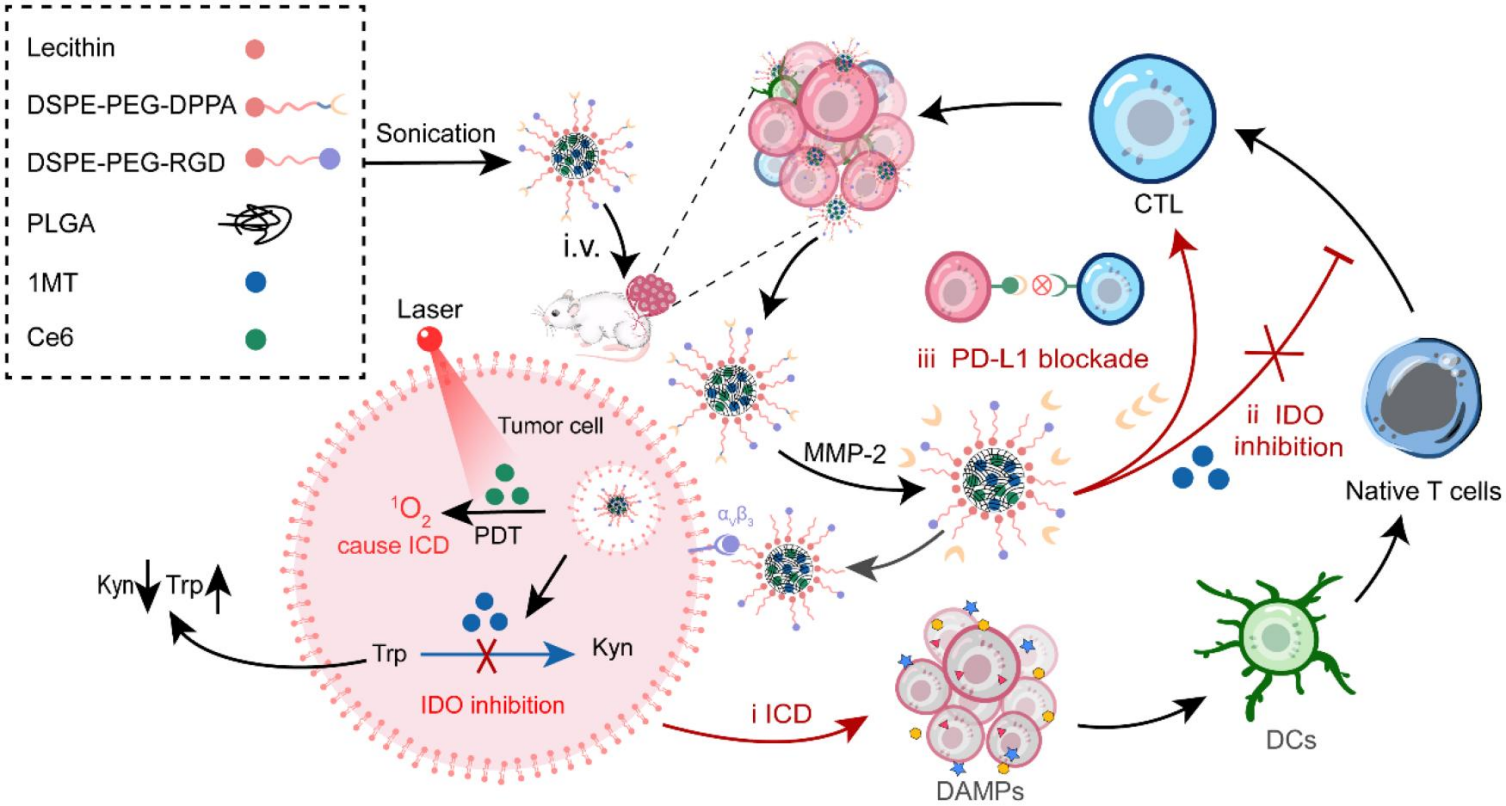
Illustration of lipid/PLGA nanocomplex activated antitumor immune effect through multiple synergistic strategies.

Herein, a multi-functional lipid/polymer nanocomplex was developed with simultaneous inhibition of IDO and PD-L1 to enhance the photoimmunotherapy of colon cancer. Both photosensitizer Ce6 and IDO inhibitor 1MT were encapsulated in lipid/PLGA nanocomplex (RDCM), where DSPE lipid was conjugated to peptide RGD and PD-L1 inhibitor DPPA to endow tumor targeting and PD-L1 blockade (Figure 1). Upon reaching tumor sites, RDCM could activate anti-tumor immunity through multiple synergistic strategies: the PDT effect of Ce6 induced tumor cells ICD, thus promoting the activation of CTLs while killing tumor cells. DPPA was specifically cleaved from RDCM in response to matrix metalloproteinase-2 (MMP-2) that was overexpressed in the extracellular matrix of cancer cells to block PD-L1, DPPA together with IDO inhibition via 1MT alleviated the immunosuppression of CTLs by TME. Therefore, the antitumor immune effect was significantly activated and the photoimmunotherapy efficacy of PDT was amplified remarkably. *In vivo* antitumor studies demonstrated the superior antitumor effect of RDCM, which facilitated the maturation of dendritic cells, inhibited the activity of IDO, and increased the proportion of tumor-infiltrating CTLs in CT26 tumor-bearing mice. The tumor re-challenge study proved that RDCM induced strong immune memory to prevent tumor metastasis.

## Materials and methods

### Materials

DPPA (sequence: CPLGVRGK-GGG-d(NYSKPTDRQYHF)) and RGD (sequence: Cyclo (RGDd(Y)KC)) were purchased from APeptide (Shanghai, China). 1,2-Distearoyl-snglycero-3-phosphoethanolamine-N-methoxy (poly (ethylene glycol)-2000) (DSPE-PEG) and 1,2-distearoyl-sn-glycero-3-phosphoethanolamine-N-maleimide (poly (ethylene glycol)-2000) (DSPE-PEG-Mal) were purchased from Laysan Bio, Inc (AL 35016, Arab). Poly (DL-lactic-co-glycolic acid) (PLGA), DSPE-PEG-RGD and DSPE-PEG-DPPA were synthesized according to our previous study [32, 36]. 1-Methyl-D-tryptophan (1MT) was obtained from Selleck (Shanghai, China). L-Kynurenine and L-tryptophan were purchased from Makclin Biochemical Co., Ltd (Shanghai, China). Chlorin e6 (Ce6) and lecithin were purchased from Aladdin Bio-Chem Technology Co., Ltd (Shanghai, China). Roswell Park Memorial Institute 1640 medium (RPMI) was obtained from HyClone (USA). Fetal bovine serum (FBS) was purchased from Gibco Life Technologies (USA). Singlet Oxygen Sensor Green (SOSG) probe was from Meilun Biotechnology (Dalian, China). Reactive oxygen species assay kit, Annexin V-FITC apoptosis detection kit, and enhanced ATP assay kit were obtained from Beyotime Biotechnology (Shanghai, China). Rabbit anti-HMGB1 and rabbit anti-CRT were from Abcam (UK) or R&D systems (USA). Recombinant human MMP-2 was obtained from PeproTech, Inc. (USA). FITC anti-mouse CD11c, BV421 anti-mouse CD86, APC anti-mouse CD80, FITC anti-mouse CD3ε, Percp-cy5.5 anti-mouse CD8, BV605 anti-mouse CD4 and PE anti-mouse Foxp3 were purchased from BD Biosciences (USA) or Biolegend (USA).

### MMP-2 cleavage activity of DSPE-PEG-DPPA

DSPE-PEG-DPPA and MMP-2 were prepared as a mixed solution (DSPE-PEG-DPPA: 3 mg/ml, MMP-2: 200 ng/ml) in trehalose buffer, incubated (37°C, 2 h) and analyzed through MALDI-TOF.

### Preparation of drug-loaded nanocomplexes

Nanoparticles were prepared using a single-step sonication method [37]. PLGA (2.5 mg/ml), Ce6 (2.5 mg/ml) and 1MT (1 mg/ml) were dissolved in DMSO, respectively. Lecithin (2 mg/ml) and DSPE-PEG (1 mg/ml) were dissolved in 4% (v/v) ethanol–water solution. PLGA, Ce6 and 1MT at different feeding ratios were mixed and incubated (2 h, 37°C). After incubation, the mixed solution was added drop-wise to the mixed solution of lecithin and DSPE-PEG. PLGA:Lecithin:DSPE-PEG was 1:0.1:0.15 (mass ratio), and the volume of water was adjusted to 10 times that of the organic solvent. Then the mixed solution was sonicated for 90 s using a probe sonicator (20 kHz, 130 W). The nanoparticle (CM) suspension was washed through Amicon Ultra-4 centrifuge filters (MWCO 10 kDa) to remove organic solvents. Nanoparticles modified with RGD (RCM, PLGA:Lecithin:DSPE-PEG:DSPE-PEG-RGD was 1:0.1:0.05:0.1 (mass ratio)) or RGD and DPPA (RDCM, PLGA:Lecithin:DSPE-PEG-DPPA:DSPE-PEG-RGD was 1:0.1:0.05:0.1 (mass ratio)) were prepared in a similar method.

The concentration of Ce6 and 1MT encapsulated in nanoparticles were detected as follows. The nanoparticles aqueous solution was centrifuged (16 000 r/min, 20 min) and the Ce6 in supernatant was detected by UV-vis spectroscopy (660 nm). And 1MT concentration was performed through high performance liquid chromatography (HPLC) [38, 39]. The drug loading content (DLC) was analyzed according our previously reports [32].

### Stability test

Nanocomplexes were suspended in PBS, H_2_O or 10% FBS at 24°C. The dynamic light scattering (DLS) size and polydispersity (PDI) were measured at different time points using a Zetasizer (Malvern Nano-ZS90).

### *In vitro* detection of singlet oxygen (^1^O_2_)

The nanocomplexes aqueous suspension containing 2 µM SOSG probe was irradiated by a laser (660 nm, 1.0 W/cm^2^) for different times. Fluorescence spectrum was recorded (Ex: 504 nm) with a spectrophotometer (F-7000 FL, Hitachi).

### Cell culture

The mouse colon cancer cells (CT26) were cultured in RPMI 1640 medium containing 10% FBS, 100 U/ml penicillin, and 100 U/ml streptomycin in a 5% CO_2_ incubator at 37°C.

### Cytotoxicity

Cells were incubated with different concentrations of Ce6 + 1MT, CM, RCM, and RDCM, and the mass ratio of Ce6 to 1MT was 3:2. After 4 h, cells were irradiated by a laser (660 nm, 1.0 W/cm^2^, 30 s). The cell viability was assessed by a CCK8 assay [40–43].

### Cellular uptake

Cellular internalization of nanocomplexes was assessed with confocal laser scanning microscopy (CLSM) and flow cytometry (FCM). For CLSM study, Ce6 + 1MT, CM, RCM and RDCM (Ce6: 3 µg/ml, 1MT: 2 µg/ml) were added to treat with cells. After a 1- or 4-h incubation, cells were stained with Hoechst 3342, and observed using CLSM. Then cells were incubated with the same concentration of nanoparticles, digested, washed with PBS and detected by FCM. To analyze targeting efficiency of RGD, cells were cultured with medium containing 20 µM RGD for 1 h, and then treated with CM, RCM and RDCM for another 1 h. Cells incubated with nanoparticles without RGD pre-incubation were set as a control. Finally, cells were suspended in PBS for FCM analysis.

### Detection of intracellular ROS

The levels of ROS produced by cancer cells were detected by DCFH-DA probes. Briefly, CT26 cells were treated with Ce6 + 1MT, CM, RCM and RDCM for 4 h (Ce6: 15 µg/ml, 1MT: 10 µg/ml), then cultured with DCFH-DA containing, serum-free medium, and performed by CLSM after irradiation (660 nm, 1.0 W/cm^2^, 3 min). The FCM study was similar to that of CLSM.

### Apoptosis study

CT26 cells were incubated with Ce6 + 1MT, CM, RCM and RDCM (Ce6: 4.5 µg/ml, 1MT: 3 µg/ml). After 4 h, the cells were irradiated with a 660 nm laser for 3 min. Then the cells were further cultured for 6 h and stained with annexin V/PI assay kit for FCM study.

### Induction of ICD *in vitro*

ICD markers CRT and HMGB1 were detected by immunofluorescence staining. Firstly, CT26 cells were cultured with Ce6 + 1MT, CM, RCM and RDCM for 4 h (Ce6: 4.5 µg/ml, 1MT: 3 µg/ml). Then cells received an irradiation (660 nm, 1.0 W/cm^2^, 3 min) and were cultured for another 6 h. The cells were fixed and blocked with 4% paraformaldehyde and 10% goat serum solution, and stained with Rabbit anti-CRT antibody, FITC-Goat anti-Rabbit IgG antibody, Hoechst 3342, and observed by CLSM. As HMGB1 was expressed intracellularly, cells were treated with Triton X after fixation with 4% paraformaldehyde. Other procedures were similar to CRT evaluation. For the ATP assay, cells were treated in a similar procedure, and the culture medium was collected after laser treatment. The concentration of ATP was investigated with an ATP assay kit.

### *In vitro* inhibition of IDO

CT26 cells were incubated with Ce6 + 1MT, CM, RCM and RDCM (Ce6: 0.03 mg/ml, 1MT: 0.02 mg/ml) for 4 h, and simultaneously treated with 0.05 µg/ml IFN-γ to induce the expression of IDO [7, 44]. After incubation, 0.15 ml supernatant was mixed with 10 µl 30% trichloroacetic acid and then the mixed solution was centrifuged (12 000 *g*, 10 min); the concentration of Kyn in the supernatant was detected by HPLC [45, 46].

### *In vitro* affinity for PD-L1

CT26 cells were blocked with 10% FBS solution. RDCM was treated with 0.2 μg/ml MMP-2, after 2 h the mixed solution was used to treat the cells for 4 h. The cells were then treated with biotinylated mPD-1 for 4 h and stained with APC streptavidin for 30 min to identify PD-1, and the cells were detected by FCM.

### Fluorescence imaging *in vivo* and *in vitro*

*In vivo* distribution was evaluated by nanoparticles loaded with infrared dye 1,1-dioctadecyl-3,3,3,3-tetramethylindocarbocyanine perchlorate (DiD). CT26 tumor-bearing mice were administrated with DiD and RDCM@DiD (DiD: 2 mg/kg) and were intravenously injected via tail vein (*n *=* *3). Fluorescence signals were analyzed by IVIS spectrum system (In Vivo lmaging System, PerkinElmer Lumina III) at preset time points. The tumors and major organs were excised 36 h after injection for fluorescence imaging.

### *In vivo* antitumor study

All animal experiments were approved by the Medical Ethics Committee of Sichuan University with an approval ID (KS2023419). CT26 cell suspension (1 × 10^6 ^cells/ml, 50 µl) was injected into the right back of 6-week-old Balb/c mice. When the tumor volume reached about 100 mm^3^, the mice were divided into six groups at random: Saline, Ce6, 1MT, Ce6 + 1MT, CM and RDCM, respectively. Nanoparticles were administered intravenously, at a dosage of 3, 2 and 2 mg/kg for Ce6, 1MT and DPPA, respectively. Mice in groups Ce6, Ce6 + 1MT, CM and RDCM received irradiation (660 nm, 1.0 W/cm^2^, 15 min) 12 h after injection. The body weight and tumor volume of mice were observed during treatment. The spleens of mice were collected to analyze DCs, and the tumors were used to study H&E staining, TUNEL, Ki67, and T cells [47, 48].

To evaluate the effect of RDCM against tumor metastasis, CT26 tumor-bearing mice were injected with Saline, Ce6 + 1MT, CM and RDCM (Ce6: 3 mg/kg, 1MT: 2 mg/kg, DPPA: 2 mg/kg) via the tail vein. After administration for 12 h, mice were irradiated with a laser (660 nm, 1.0 W/cm^2^, 15 min), and the tumors were surgically removed at day 14. After surgery, Luc-CT26 cells were injected into the rectal mucosa of mice. The bioluminescence of tumors in mice was detected by IVIS spectrum system. On day 21, the spleens of mice were used to study T cells.

### Statistical analysis

The data were presented as mean ± SD. Statistical differences were calculated using a Student’s *t*-test. *P *<* *0.05 indicated a statistically significant difference.

## Results and discussion

### Synthesis and characterization of RDCM

Nanoparticles were prepared by a one-step method, the high-energy, uniform sonication provided energy for nanoparticle formation [37]. PLGA, a biodegradable polymer approved by the FDA for medical applications, was utilized to form the core of the nanoparticles. FDA-approved polymer DSPE-PEG provided shells for nanoparticles to reduce nonspecific protein adsorption and prolong *in vivo* circulation. Natural neutral lecithin comprises a monolayer lipid layer between the core and shell to provide stability for nanoparticles and prevent drug leakage. RGD and DPPA were used to modify the nanoparticles to target tumor cells and block PD-L1, respectively. PLGA was synthesized by ring-opening polymerization [36], DSPE-PEG-RGD and DSPE-PEG-DPPA were synthesized by click reaction of maleimide in DSPE-PEG-Mal with sulfhydryl in RGD or DPPA according to our previous study [32]. The MMP-2 cleavage activity of DSPE-PEG-DPPA was shown in Supplementary Figure S1. DSPE-PEG-Mal was treated with MMP-2 at 37°C for 2 h, and the peaks of ‘RGK-GGG-d (NYSKPTDRQYHF)’ (2168.7) and ‘DSPE-PEG-CPLG’ (2389) cleaved from DSPE-PEG-DPPA were closed to the theoretical prediction of 2167.3 Da and 2388 Da, respectively, clearly demonstrating the sensitivity of DSPE-PEG-DPPA to MMP-2.

**Figure 2.**
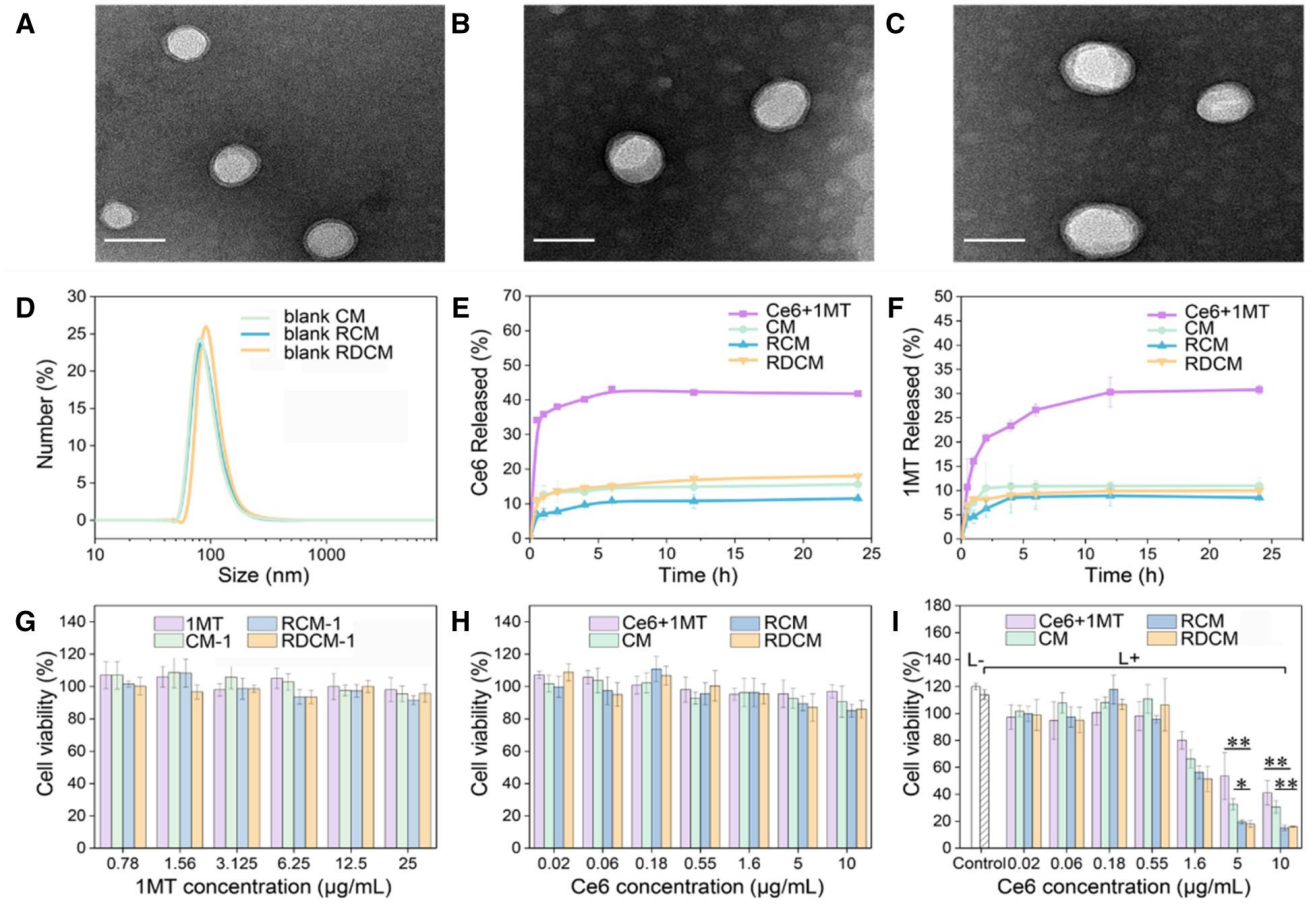
TEM images of CM (**A**), RCM (**B**) and RDCM (**C**); scale bars represent 50 nm. DLS sizes of blank CM, RCM and RDCM (**D**). The release curves of Ce6 (**E**) and 1MT (**F**) *in vitro*. Cell viability of CT26 cells incubated with 1MT, CM-1, RCM-1 and RDCM-1 (**G**), Ce6 + 1MT, CM, RCM and RDCM for 48 h (**H**). Cell viability of CT26 cells incubated with Ce6 + 1MT, CM, RCM and RDCM for 24 h after 660 nm laser irradiation for 30 s (**I**) (*n *=* *3, *P <* *0.05, **P <* *0.01).

Illustrations of CM (nanoparticle without surface modification), RCM (RGD modified nanoparticles), and RDCM (RGD and DPPA modified nanoparticles) were shown in Supplementary Figure S2. Transmission electron microscopy (TEM) results illustrated that the nanoparticles were spherical with a core-shell structure (Figure 2A–C), and the particle size was about 50 nm. The hydrodynamic diameters of blank nanoparticles were 94, 98 and 106 nm (Figure 2D), respectively, the diameters of nanoparticles tested by DLS were larger than those in TEM results probably because TEM images exclusively captured the dehydrated morphology of nanoparticles, while DLS provided the hydrodynamic diameters of nanoparticles in aqueous suspensions. The zeta potentials of blank CM, RCM and RDCM were −15.3, −16.6 and −14.1 mV, respectively (Supplementary Figure S3A).

### *In vitro* drug release and singlet oxygen (^1^O_2_) generation

1MT and Ce6 encapsulated in nanoparticles through hydrophobic interactions. DLCs of Ce6 and 1MT with different feeding ratios of nanoparticles were shown in Supplementary Table S1. The DLCs of Ce6 and 1MT increased with the increase of the feeding amount. To ensure the therapeutic effect of Ce6 and 1MT, the mass ratio of Ce6 and 1MT was set to 3:2. The hydrodynamic diameter of the drug-loaded CM, RCM, and RDCM were 121, 125 and 138 nm (Supplementary Figure S3B), which were slightly larger than those of the blank nanoparticles. There was no significant difference in zeta potential between drug-loaded nanoparticles and blank nanoparticles (Supplementary Figure S3A). The UV-vis absorptions of three nanoparticles were shown in Supplementary Figure S3C. After loaded with Ce6 and 1MT (Ce6: 15 µg/ml, 1MT: 10 µg/ml), the three nanoparticles showed strong UV-vis absorption at 665 nm, it indicated that Ce6 was successfully encapsulated for PDT. Stability is a crucial characteristic of nanoparticles. As displayed in Supplementary Figure S4, the size and PDI of the three nanoparticles were stable within 24 h in 10% FBS solution and 48 h in H_2_O and PBS.

The release curves of Ce6 showed that the cumulative release of the three nanoparticles was similar (Figure 2E). The cumulative releases of Ce6 were 15.6% (CM), 11.5% (RCM) and 18% (RDCM) at 24 h. The release of 1MT (Figure 2F) was consistent with that of Ce6, and the cumulative releases of 1MT were about 11% (CM), 8.5% (RCM) and 10% (RDCM) at 24 h. The reason why the cumulative release amount of Ce6 was higher than that of 1MT was probably that the hydrophilicity of Ce6 was stronger than that of 1MT.

Ce6 as a photosensitizer could generate ^1^O_2_ under 660 nm laser irradiation. SOSG probe was utilized to characterize the ^1^O_2_ generated after the nanoparticles were irradiated by a 660 nm laser. The fluorescence intensity at 525 nm was positively correlated with the concentration of ^1^O_2_. After incubation with RDCM, the fluorescence intensity of SOSG increased with the prolongation of irradiation time (Supplementary Figure S3D), implying the promising ^1^O_2_ generation ability of RDCM. The results of CM and RCM were similar to those of RDCM.

### *In vitro* cytotoxicity

Before analyzing the antitumor effect of drug-loaded nanoparticles, the drug cytotoxicity was studied. As shown in Figure 2G, when the concentration reached 25 µg/ml, 1MT displayed almost no toxicity after incubated with CT26 cells for 48 h. This was due to the fact that 1MT was an IDO inhibitor, which activated the immune system by inhibiting the activity of IDO but not eliciting strong cytotoxicity [7]. Similarly, 1MT single-loaded nanoparticles: CM-1, RCM-1 and RDCM-1 displayed almost no toxicity to CT26 cells. The cytotoxicity of free Ce6 + 1MT and co-loaded Ce6 + 1MT nanoparticles CM, RCM and RDCM to CT26 cells was evaluated with 24 h incubation (Figure 2H). When the concentration of Ce6 reached 10 µg/ml, the cell viability of CM, RCM and RDCM were more than 80%, suggesting the negligible toxicity of the three nanoparticles. The *in vitro* anticancer activity of nanoparticles with irradiation was shown in Figure 2I. When the concentration of Ce6 increased, the cell viability of each group decreased gradually. The cell viability of Ce6 + 1MT, CM, RCM and RDCM groups were 53.5%, 32.6%, 19.4% and 17.9% (Ce6 5 µg/ml), respectively. The cell viability of CM, RCM and RDCM were significantly lower than that of Ce6 + 1MT, indicating the therapeutic enhancement of PDT. The cell viability of RCM and RDCM was significantly lower than that of CM due to the promotion internalization effect of RGD. The cell viabilities of RCM and RDCM were similar, revealing no significant impact of DPPA modification on PDT therapeutic effect.

### Cellular uptake, ROS generation, apoptosis study

**Figure 3.**
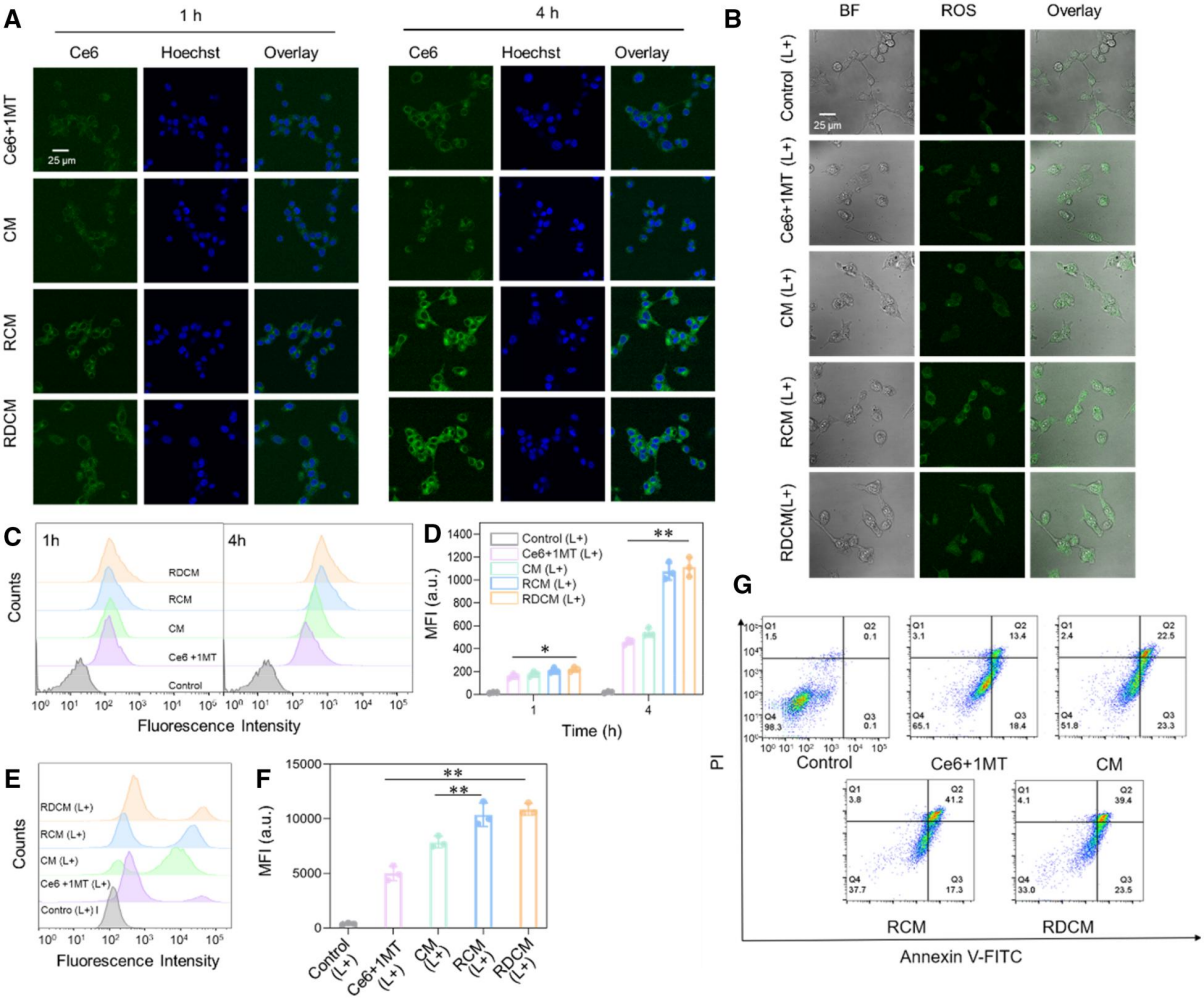
CLSM images and FCM results of cellular uptake (**A**, **C** and **D**) and intracellular ROS (**B**, **E** and **F**) (*n *=* *3, *P <* *0.05, **P <* *0.01). Apoptosis rates of cells treated with Ce6 + 1MT, CM, RCM and RDCM after 660 nm laser irradiation (**G**).

CLSM and FCM were applied to qualitatively and quantitatively measure the fluorescence intensities of Ce6 to study the internalization of nanoparticles. The CLSM results were shown in Figure 3A. After incubation for 1 and 4 h, the fluorescence intensity of Ce6-loaded nanoparticles was time-dependent, and remarkably stronger than that of free drug combination (Ce6 + 1MT). Moreover, the fluorescence signals of RCM and RDCM were stronger when compared with CM. The results of FCM were shown in Figure 3C and D. When the cells were treated for 4 h, the mean fluorescence intensity (MFIs) of CM, RCM and RDCM were 1.2, 2.3 and 2.2 times higher than those of the Ce6 + 1MT group, suggesting that the nanoparticles facilitated the internalization of Ce6. Moreover, the MFIs of RCM and RDCM groups were 1.9 and 1.7 times higher than that of CM, which were consistent with the CLSM results and MTT, demonstrating that RGD contributed to the internalization of nanoparticles.

To further study the targeting efficiency of RGD, CT26 cells were pre-treated with RGD for 1 h, and then incubated with RCM and RDCM for 1 h. The cells incubated with RCM and RDCM without RGD pre-incubation were set as the positive control and the fluorescence intensity of Ce6 in each group was analyzed by FCM (Supplementary Figure S5). After RGD pre-incubation, the fluorescence intensity of RDCM was reduced obviously, indicating that the enhanced cellular uptake of Ce6 was due to the specific binding between RGD and integrins highly expressed on the surface of CT26 cells.

Intracellular ROS levels were measured by DCF-DA probe. DCFH-DA could form DCF with green fluorescence in response to ROS [49–51]. The fluorescence intensity of DCF was qualitatively and quantitatively detected by CLSM and FCM. The CLSM results of intracellular ROS illustrated that the fluorescence intensities of RCM and RDCM were the strongest, followed by CM, and the weakest in the Ce6 + 1MT group (Figure 3B). The results of FCM (Figure 3E and F) and CLSM were consistent, confirming the strongest PDT effect of RCM and RDCM.

Apoptosis rates of cells treated with nanoparticles were assessed by an annexin V/PI assay kit (Figure 3G). The apoptosis rates of cells treated with RCM and RDCM were as high as 41.2% and 39.4%, and that of CM group was 22.5%, these results were consistent with the results of cytotoxicity, cellular internalization, and intracellular ROS evaluation, proving that the RCM and RDCM with RGD modification could promote the cellular uptake of Ce6 to generate more ROS and lead to more cells apoptosis, resulting in a superior antitumor effect *in vitro*.

### Induction of ICD, inhibition of IDO and affinity for PD-L1 *in vitro*

**Figure 4.**
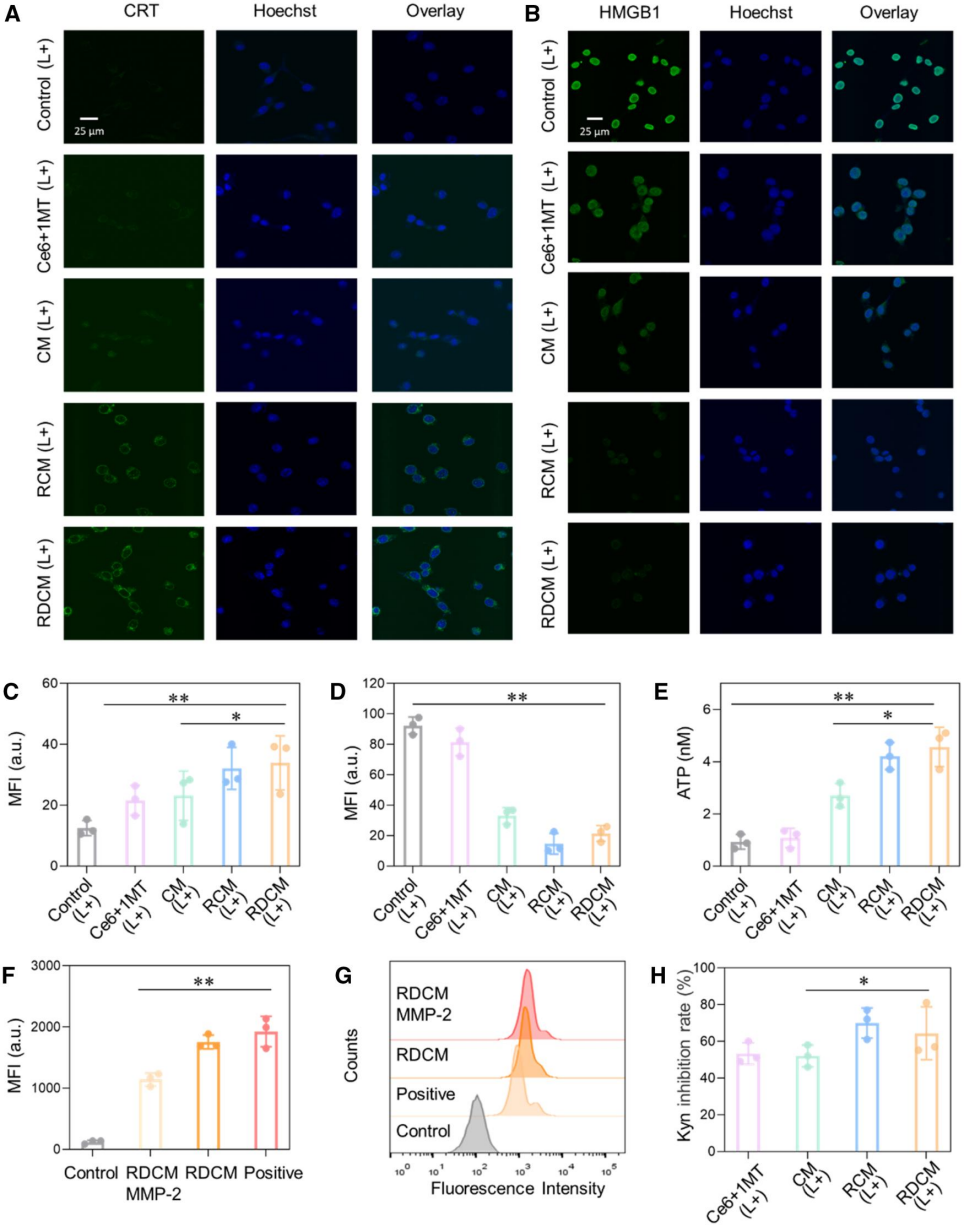
CLSM images and semi-quantitative of CRT (**A**, **C**) and HMGB1 (**B**, **D**) of CT26 cells treated with Ce6 + 1MT, CM, RCM and RDCM after 660 nm laser irradiation. ATP released from CT26 cells treated with Ce6 + 1MT, CM, RCM and RDCM after 660 nm laser irradiation (**E**). Affinity detection of RDCM to PD-L1 after pretreatment with MMP-2 (**F** and **G**). Kyn inhibition rates of CT26 cells (**H**). *n *=* *3, *P <* *0.05, **P <* *0.01.

Studies have demonstrated that tumor cells killed by PDT would release DAMPs (CRT, HMGB1, ATP, etc.) to conducive the activation of the immune system [19, 52]. The CRT (Figure 4A and C) and ATP levels (Figure 4E) of cells incubated with RCM and RDCM were the highest, while the HMGB1 level was the lowest (Figure 4B and D). It revealed that intracellular HMGB1 was released during ICD, indicating the optimal ICD induction effect of RCM and RDCM.

IDO-overexpressing tumor cells catalyzed Trp to Kyn, leading to Trp depletion and Kyn accumulation and resulting in tumor immune escape [39]. The concentration of Kyn in the medium with Ce6 + 1MT, CM, RCM and RDCM incubation for 48 h was detected by HPLC to investigate the influence of RGD on the therapeutic effect of 1MT. The inhibitory ability of 1MT on IDO activity was positively correlated with the concentration [7, 8]. Combined with the DLC of nanoparticles to 1MT, the concentration was set to 20 μg/ml for this study. As shown in Figure 4H, the inhibition rates of Kyn in the 1MT and CM were similar, and those in the RCM and RDCM were higher, which were 1.2 and 1.15 times of the CM group, indicating that RGD promoted the IDO inhibiting effect of 1MT.

DPPA could be cleaved from DSPE-PEG-DPPA through MMP-2, we analyzed whether DPPA could block the interaction between PD-1 and PD-L1 *in vitro*. CT26 cells over-expressing PD-L1 [20, 53] were incubated with RDCM pre-incubated with MMP-2, and mPD-1 was added to simulate the binding of T cells and cancer cells. FCM results (Figure 4F and G) demonstrated that the MFI of RDCM pre-incubated with MMP-2 was 0.5 times to that of the positive group, implying the PD-L1 blockade effect of DPPA.

### Fluorescence imaging *in vivo* and *ex vivo*

The biodistribution of nanoparticles was studied. RDCM was labeled with DiD (a near-infrared dye), and observed by IVIS spectrum system. The fluorescence of tumor in RDCM group increased gradually and reached the maximum at 12 h. Although the DiD group also showed fluorescence in the tumor site, it was observed in the body (Supplementary Figure S6A). Mice were sacrificed at 36 h, and the *ex vivo* fluorescence images of the main organs and tumors (Supplementary Figure S6B), and semi-quantitative of the fluorescence signal (Supplementary Figure S6C) were studied. The strong fluorescence was observed in the tumor in RDCM group, and the MFI was 1.8 times that of DiD group, indicating the accumulation of RDCM in the tumor with the targeting effect of RGD, which was favorable for antitumor *in vivo*.

### *In vivo* antitumor study

**Figure 5.**
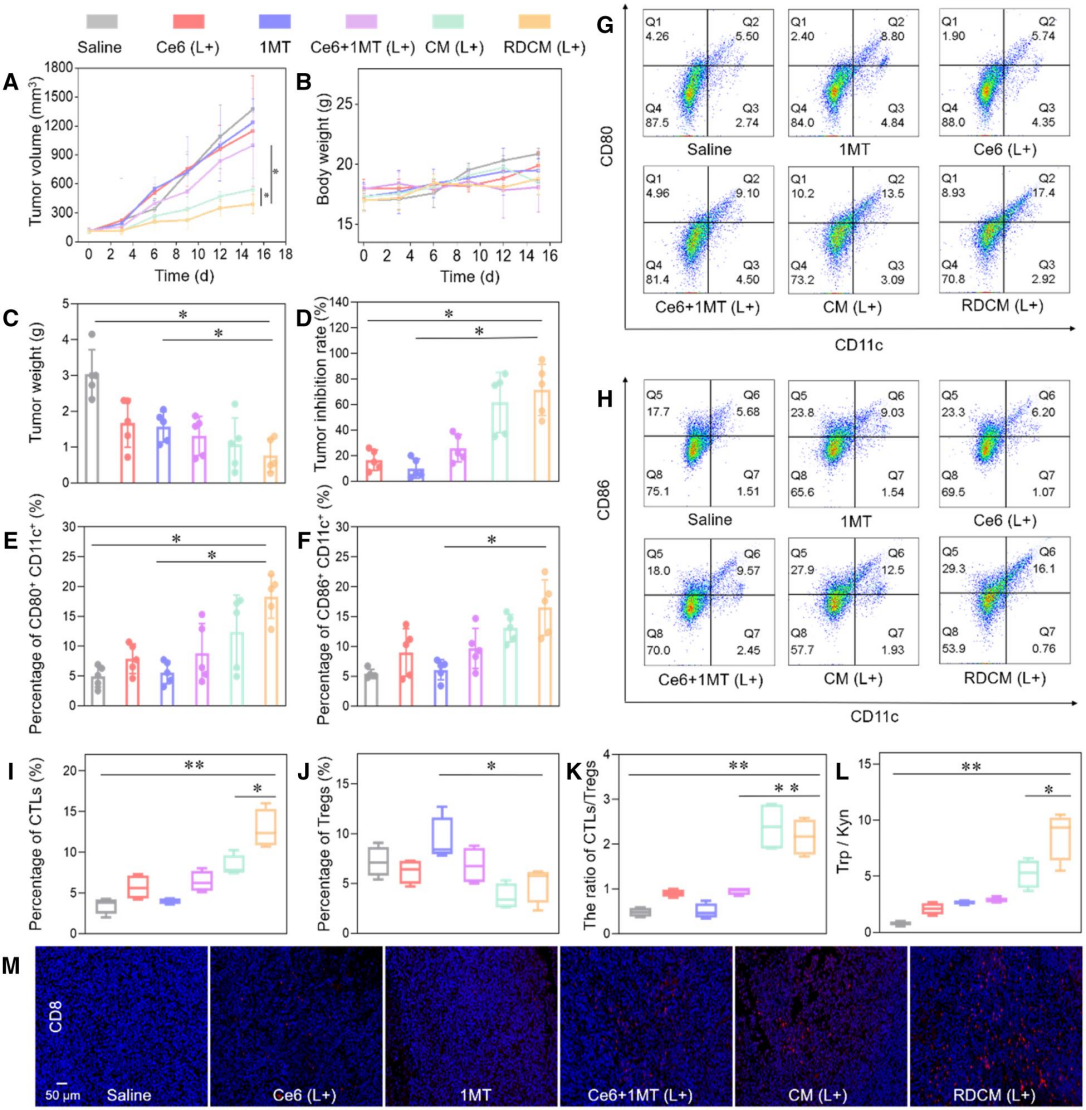
Tumor volumes (**A**), body weights (**B**), tumor weights (**C**), tumor inhibition rates (**D**) of mice in different groups, *n *=* *5, *P <* *0.05, **P <* *0.01. FCM analysis of CD11c and CD80 expression analysis (**G**), CD11c and CD86 expression analysis (**H**), and statistical results of CD11c^+^ CD80^+^ cells (**E**) and CD11c^+^ CD86^+^ cells (**F**), *n *=* *5, *P <* *0.05, **P <* *0.01. Statistical results of percentages of CTLs in T cells (**I**), percentages of Tregs in T cells in different treatment (**J**), ratios of CTLs/Treg T cells (**K**), *n *=* *4, *P <* *0.05, **P <* *0.01. The ratio of Trp/Kyn (**L**) in the serum of mice. CD8 immunofluorescence analysis of the tumor slices in different groups (**M**), *n *=* *4, *P <* *0.05, **P <* *0.01.

Mice with CT26 tumor-bearing mice were administered with Saline, Ce6, 1MT, Ce6 + 1MT, CM, and RDCM (Ce6 3 mg/kg, 1MT 2 mg/kg and DPPA 2 mg/kg) via tail vein every two days and injected for five times. After administration for 12 h, mice in Ce6, Ce6 + 1MT, CM and RDCM groups were irradiated with 660 nm (1.0 W/cm^2^) for 15 min. Figure 5A revealed the changes of tumor volume during the treatments. Saline, 1MT and Ce6 could hardly inhibit tumor growth, which was due to the low accumulation of 1MT and Ce6 in the tumor and the relatively low dosage compared to the related studies (1MT: 5–10 mg/kg [8, 54], Ce6: 3–10 mg/kg [15, 55, 56]). The tumor volume of the CM group was 0.54 times to that of the Ce6 + 1MT group, reflecting that nanoparticles were beneficial to improve the bioavailability of drugs. And the tumor volume of the RDCM group was 0.72 times that of the CM group, indicating that PDT combined with immune checkpoint IDO and PD-L1 blockade achieved the best synergistic therapeutic effect.

After 15 days of treatment, tumor inhibition rate results were consistent with tumor weight and volume (Figure 5D). The highest tumor inhibition rate in the RDCM group was 70%, the tumor weight in the RDCM group was also the lightest at 0.73 g, which was 0.56 and 0.66 times to that of the 1MT+Ce6 and CM groups, respectively (Figure 5C). The weight of mice in each group increased with time, indicating the biosafety of nanoparticles and the combination therapy (Figure 5B). After treatment, the major organs of mice were analyzed by H&E staining in each group of mice (Supplementary Figure S7). Local hemorrhage was found in the heart of Saline, Ce6 and Ce6 + 1MT groups, and pulmonary metastases were observed in Saline and Ce6 groups. There were no obvious pathological changes in the main organs of mice in the CM and RDCM groups when compared with Saline group, implying the good biocompatibility of nanoparticles. H&E, TUNEL and Ki67 analysis of tumor tissue were shown in Supplementary Figure S8. The H&E and TUNEL study showed more serious tumor necrosis and apoptosis, and Ki67 analysis showed less tumor cell proliferation in RDCM group, further confirming the superior antitumor efficiency.

### *In vivo* immune activation

DCs were involved in promoting immune system activation. The percentages of CD11c, CD80 and CD86 (markers of mature DCs) in the spleen of mice were investigated by FCM. The percentages of CD86^+^CD11c^+^ in Saline, 1MT, Ce6 and Ce6 + 1MT groups were similar (5.6–9.5%), and the ratio of CD86^+^CD11c^+^ in RDCM group was the highest (16.1%), which was 1.68 times higher than that of Ce6 + 1MT group (Figure 5F and H). The percentage of CD80^+^CD11c^+^ cells in each group was similar to CD86^+^CD11c^+^ study (Figure 5G and E). These results revealed that RDCM not only produced a significant PDT effect, but also effectively increased the ratio of the maturation DCs. In order to explore the inhibition of IDO, we used HPLC to detect the Trp and Kyn concentration in the mice serum, and calculated the Trp/Kyn ratio (Figure 5L). The Trp/Kyn ratio of RDCM group was the highest (8.2%), which was 1.5 and 2.9 times higher than that of CM and Ce6 + 1MT groups, respectively. RDCM could successfully enhance the concentration of Trp in serum and inhibit IDO *in vivo*, which was attributed to immunosuppression reduction of CTLs by TME and activation of antitumor immune effect.

The FCM results of tumor tissue infiltrating CTLs were shown in Figure 5I. The RDCM group showed the highest CTLs ratio of 12.8%, which was 1.5, 1.8 and 3.3 times to that of CM, Ce6 + 1MT and Saline, respectively. This was also demonstrated by tumor CD8 immunofluorescence analysis (Figure 5M) with the most infiltrating CD8^+^ cells in the RDCM group. Meanwhile, tumor-infiltrating regulatory T cells (Tregs) in RDCM group were 0.7 times to those in Ce6 + 1MT group (Figure 5J). And the ratio of CTLs/Tregs was studied to evaluate immune activation, the ratio of RDCM group was 2.48 and 4.96 times to that of Ce6 + 1MT and Saline groups, respectively (Figure 5K). These results were consistent with those of DCs activation and IDO inhibition, suggesting the dual immune blockade on PD-L1 and IDO of RDCM and the ICD of tumor cells caused by PDT played a strong immune activation effect on CTLs.

Cytokines were essential in immune responses, and the levels of cytokines in serum were measured. TNF-α was a multifunctional cytokine with critical role in apoptosis and inflammatory immunity. The TNF-α concentration in RDCM group was 1.6 and 4.39 times to that of CM and Ce6 + 1MT groups (Supplementary Figure S9A). IL-6 was beneficial to the proliferation of T cells, the IL-6 concentration in RDCM group was five times to that of Ce6 + 1MT group (Supplementary Figure S9B). The high levels of TNF-α and IL-6 in serum of RDCM group further confirmed that PDT combined with blocking immune checkpoint IDO and PD-L1 successfully activated the immune system.

### The immune memory effect of inhibiting metastasis of orthotopic colon cancer model

**Figure 6.**
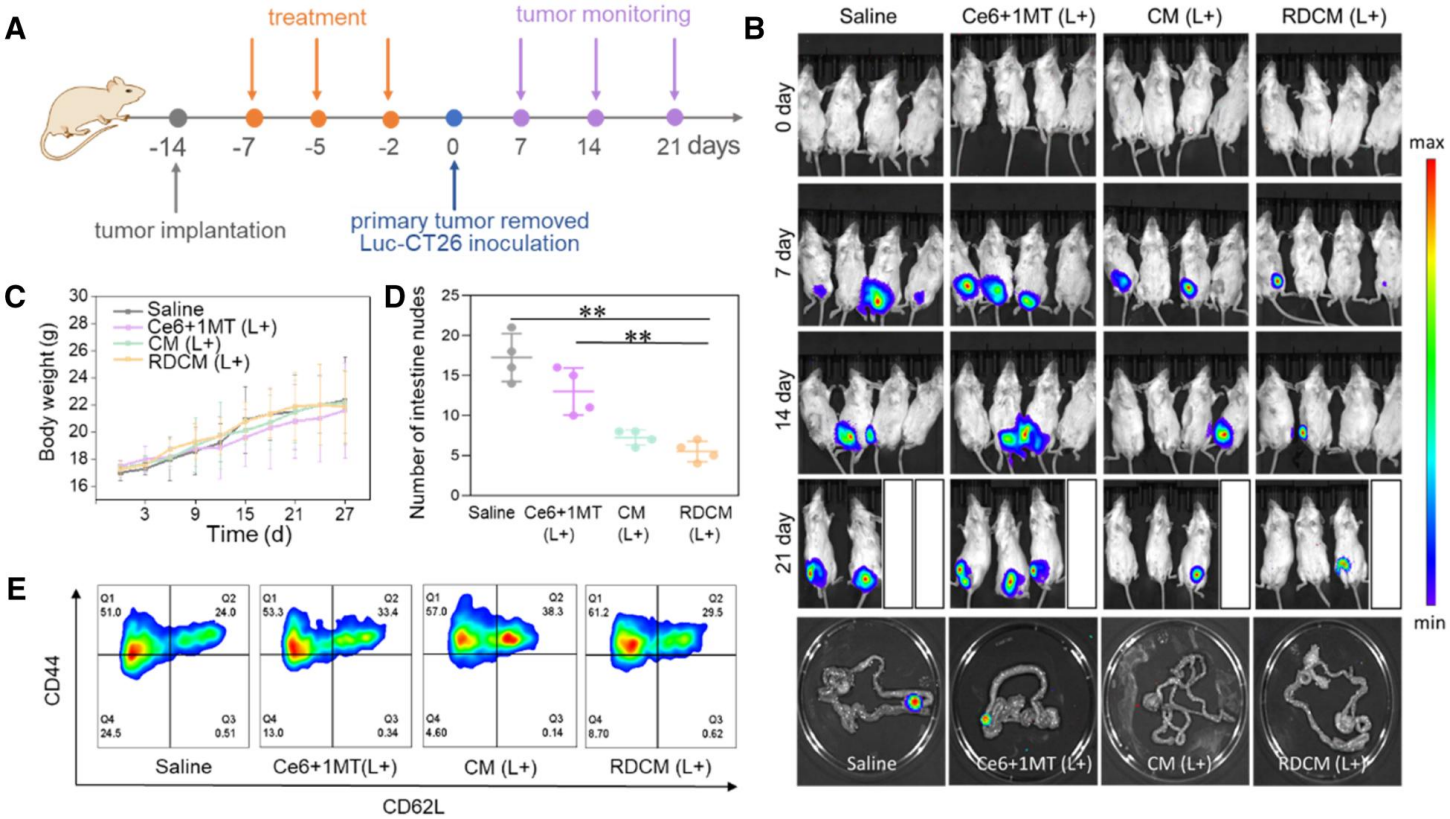
Scheme of immune memory study (**A**), *in vivo* bioluminescence images at different time points and representative intestinal bioluminescence imaging at day 21 of mice in different treatment groups (**B**). Body weight of mice during treatment (**C**). Number of intestine nudes (**D**), FCM analysis of T_EM_ in spleens (gated on CD3^+^ CD8^+^ T cells) (**E**) (*n *=* *4, *P <* *0.05, **P <* *0.01).

Encouraged by the strong anti-tumor immune effect stimulated by RDCM *in vivo*, we next examined whether RDCM could produce an immune memory effect through an orthotopic colon cancer metastasis model. CT26 subcutaneous tumor model was established, and the tumor-bearing mice were injected with Saline, Ce6 + 1MT, CM and RDCM via the tail vein (Ce6: 3 mg/kg, 1MT: 2 mg/kg, DPPA: 2 mg/kg) every two days for total three times, the scheme of immune memory study was shown in Figure 6A. After administration for 12 h, mice in Ce6 + 1MT, CM and RDCM groups were irradiated with a laser (660 nm, 1.0 W/cm^2^, 15 min). The tumor volume of RDCM group was 0.31 and 0.17 times to that of CM and Ce6 + 1MT group (Supplementary Figure S10A). And the tumor inhibition rate of RDCM group was 8.8 times to that of Ce6 + 1MT group (Supplementary Figure S10B). After the treatment, the tumor of mice was resected (Supplementary Figure S10C and D). RDCM group showed the lowest tumor mass (0.6 g).

After resection of the tumor, Luc-CT26 cells were inoculated under the rectal mucosa, and the bioluminescence was detected by IVIS spectrum system (Figure 6A). The fluorescence signal of Saline group and Ce6 + 1MT group gradually increased with time, indicating the rapid growth of tumor (Figure 6B). In contrast, the fluorescence signal of CM and RDCM groups increased slowly, and there were two mice with no obvious fluorescence in both groups on the 21st day. The intestines of the mice in each group were collected and the tumor nodules were recorded. The representative intestinal bioluminescence images of mice were shown in Figure 6B. Obvious bioluminescence can be observed in Saline and Ce6 + 1MT groups, but weaker in CM and RDCM groups, which was consistent with *in vivo* bioluminescence. The semi-quantitative of *in vivo* bioluminescence was shown in Supplementary Figure S11, the MFI of RDCM group was only 24% to that of Saline group. The tumor nudes in CM group and RDCM group were 0.45 and 0.31 times to that of Saline (Figure 6D). The above results showed that PDT combined with dual immune checkpoint blockade therapy could successfully inhibit tumor metastasis.

The body weight of mice increased steadily (Figure 6C), indicating good biosafety of RDCM. H&E analysis showed that no obvious pathological changes of major organs in mice were observed in the RDCM group (Supplementary Figure S12). To analyze the effect of immune memory in mice, FCM was used to measure the expression of CD3, CD8, CD44 and CD62L in the spleen of mice. In response to antigen stimulation, effector memory T cells (T_EM_, CD3^+^CD8^+^CD44^+^CD62L^-^) derived cytotoxic effects and produced immune memory protection [57–59]. The percentage of T_EM_ in RDCM group was the highest (61.2%), which was 1.2 times to that of Saline group (Figure 6E). These results indicated that RDCM could activate memory T cells during rechallenge tumor cells, which produced strong immune memory and inhibited tumor metastasis.

## Conclusion

In conclusion, we developed a lipid-PLGA nanoparticle (RDCM) to simultaneously inhibit IDO and PD-L1 for photodynamic and immunotherapy of colon cancer. RDCM effectively induced apoptosis of CT26 cells by PDT and ICD, inhibited the IDO activity and blocked the binding of PD-L1 to PD-1. RDCM showed excellent anti-tumor effect *in vivo*, and effectively promoted dendritic cell maturation, inhibited IDO activity, increased the proportion of tumor-infiltrating CTLs, and induced an immune memory effect to prevent tumor metastasis. The lipid-PLGA nanocomplexes provided a promising strategy for targeted photoimmunotherapy of cancer.

## Supplementary Material

rbae036_Supplementary_Data
