# Supplementary material for: A lipid/PLGA nanocomplex to reshape tumor immune microenvironment for colon cancer therapy
Source: Regen Biomater. 2024 Mar 28;11:rbae036. doi: 10.1093/rb/rbae036 (PMC11018539; doi:10.1093/rb/rbae036)
Supplement: rbae036_Supplementary_Data [file rbae036_supplementary_data.docx]

Supporting information

A Lipid/PLGA Nanocomplex to Reshape Tumor Immune Microenvironment for Colon Cancer Therapy

Nan Zhang^a, c^, Qiqi Sun^c^, Junhua Li^c^, Jing Li^c^, Lei Tang^c^, Quan Zhao^c^, Yuji Pu^c^, Gaofeng Liang^a^, Bin He^c*^, Wenxia Gao^d^, Jianlin Chen^b*^

^a^ Henan Academy of Sciences, Zhengzhou 450046, China

^b^ School of Laboratory Medicine, Sichuan Provincial Engineering Laboratory for Prevention and Control Technology of Veterinary Drug Residue in Animal-origin Food, Chengdu Medical College, Chengdu 610500, China

^c^ National Engineering Research Center for Biomaterials, College of Biomedical Engineering, Sichuan University, Chengdu 610064, China

^d^ School of Pharmacy, Chengdu University, Chengdu 610106, China

*E-mails of corresponding authors: Dr. Bin He, [bhe@scu.edu.cn](mailto:bhe@scu.edu.cn); Dr. Jianlin Chen, [chenjianlin@cmc.edu.cn](mailto:chenjianlin@cmc.edu.cn)


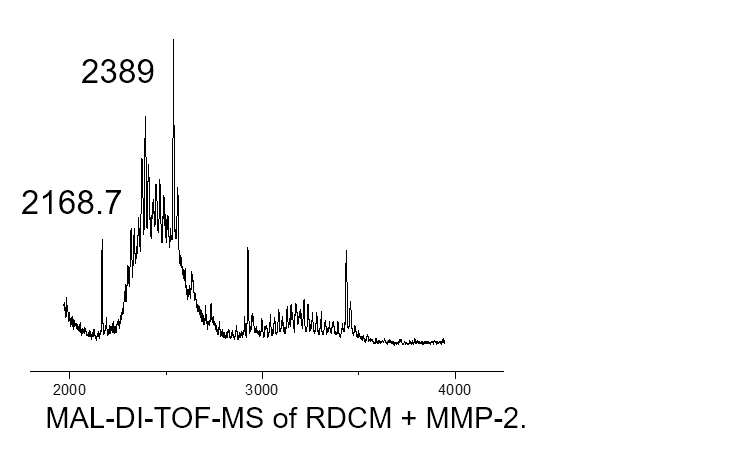


**Figure S1.** The MALDI-TOF-MS spectrum of DSPE-PEG-DPPA incubated with MMP-2.


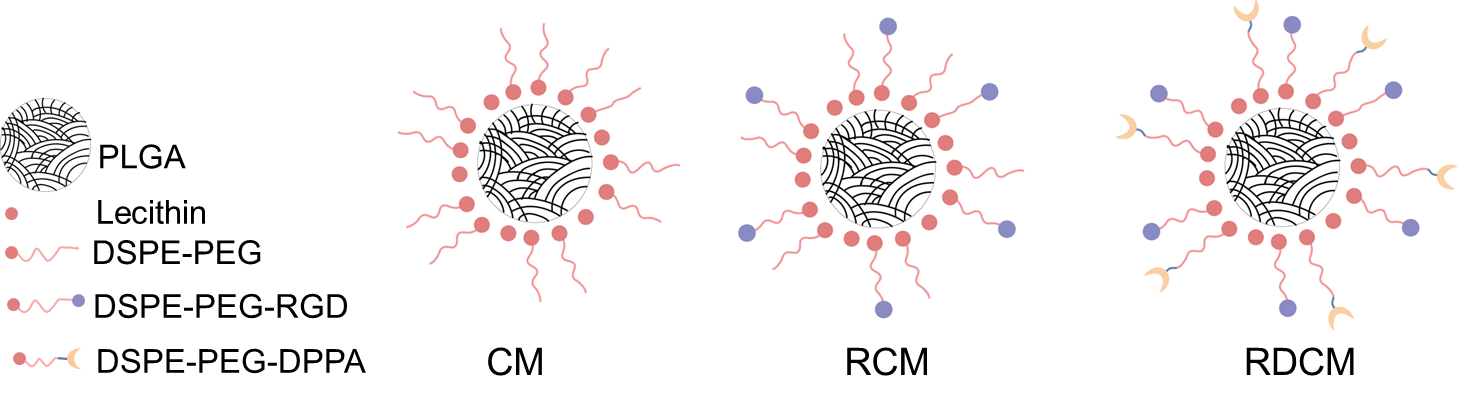


**Figure S2.** Illustrations of CM, RCM and RDCM.


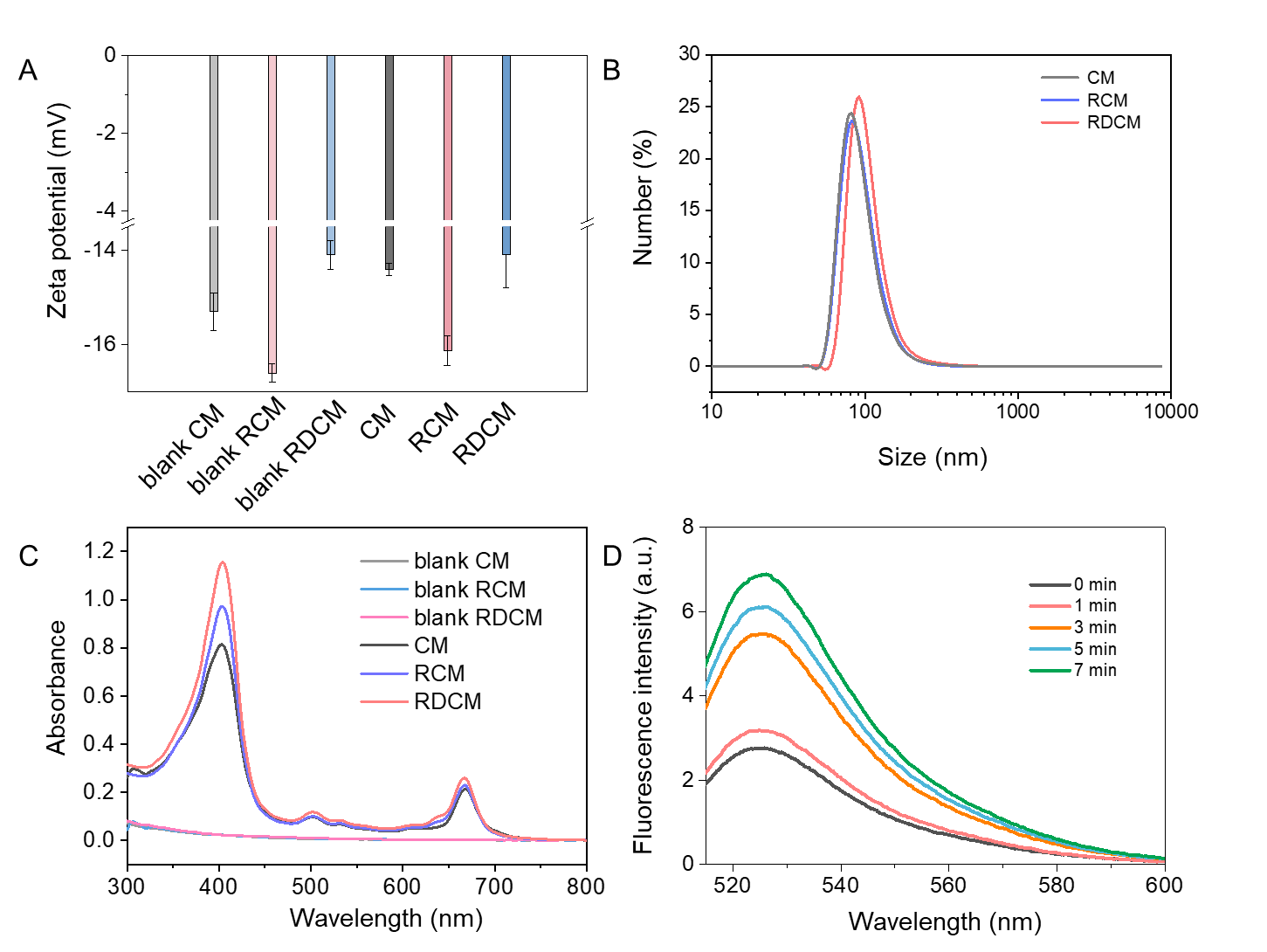


**Figure S3.** Zeta potentials of blank CM, blank RCM, blank RDCM, CM, RCM and RDCM (A). DLS sizes of CM, RCM and RDCM (B). UV-vis absorption of nanoparticles (C). Fluorescence intensity of RDCM incubated with SOSG upon different laser irradiation times (D).


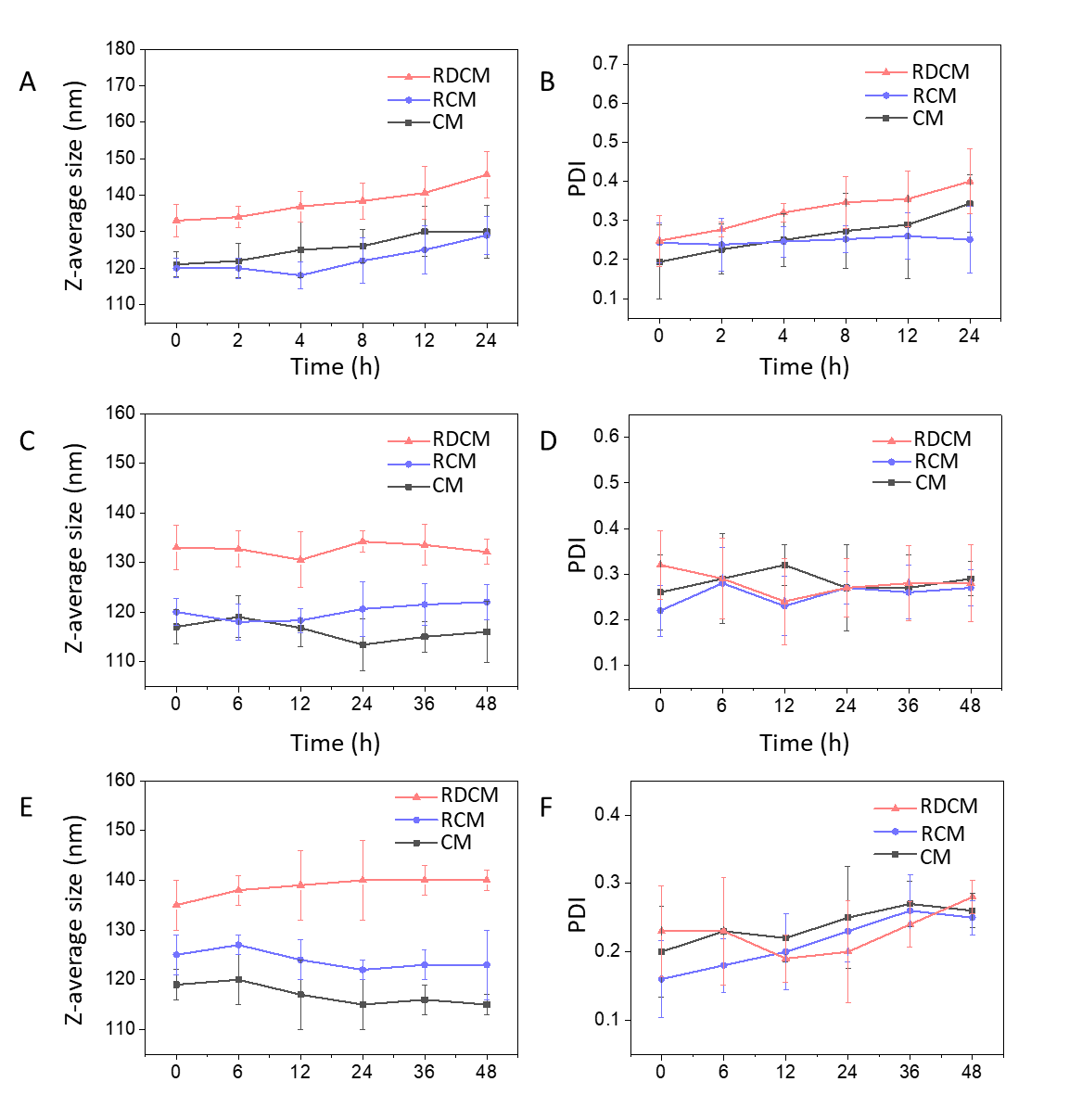


**Figure S4.** Size (A, C and E) and PDI (B, D and F) variations of CM, RCM and RDCM incubated with 10% FBS (A, B), H_2_O (C, D) and PBS (E, F).


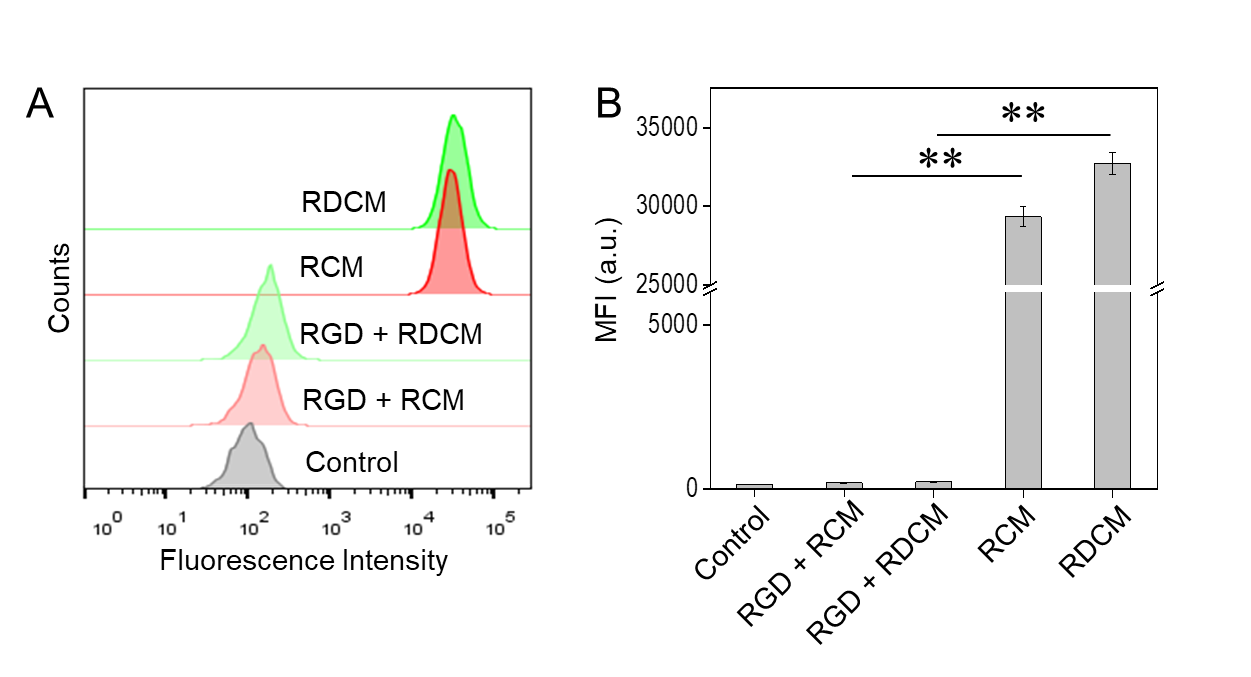


**Figure S5.** FCM analysis (A) and statistical results (B) of CT26 cells incubated with RCM and RDCM for 1 h after being treated with or without RGD (*n* = 3, **p*<0.05, ***p*<0.01).


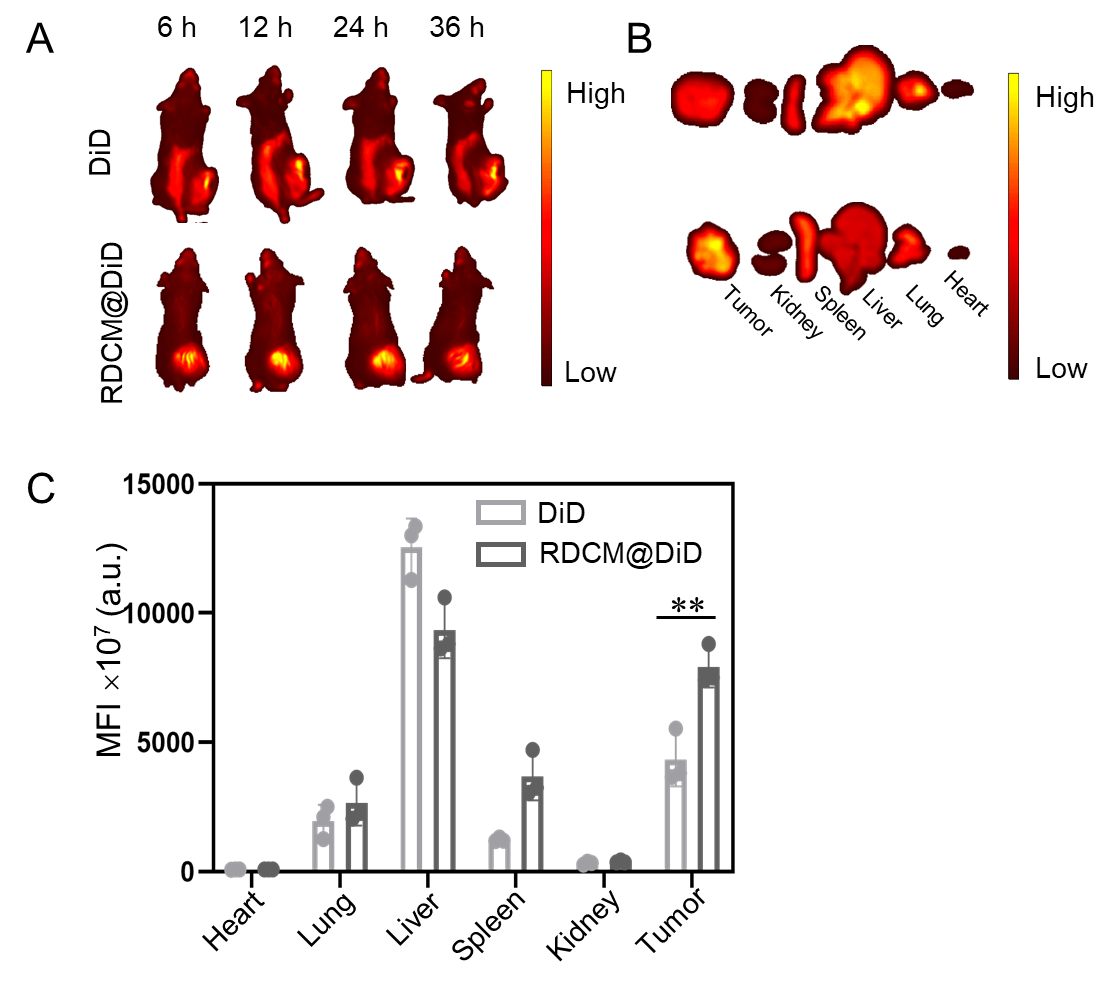


**Figure S6.** In vivo fluorescence images at different time points (A), in vitro fluorescence signal (B) and semi-quantitative analysis (C) of fluorescence signal in organs and tumors of CT26 tumor-bearing mice after DiD and RDCM@DiD administration (*n* = 3, **p*<0.05, ***p*<0.01).


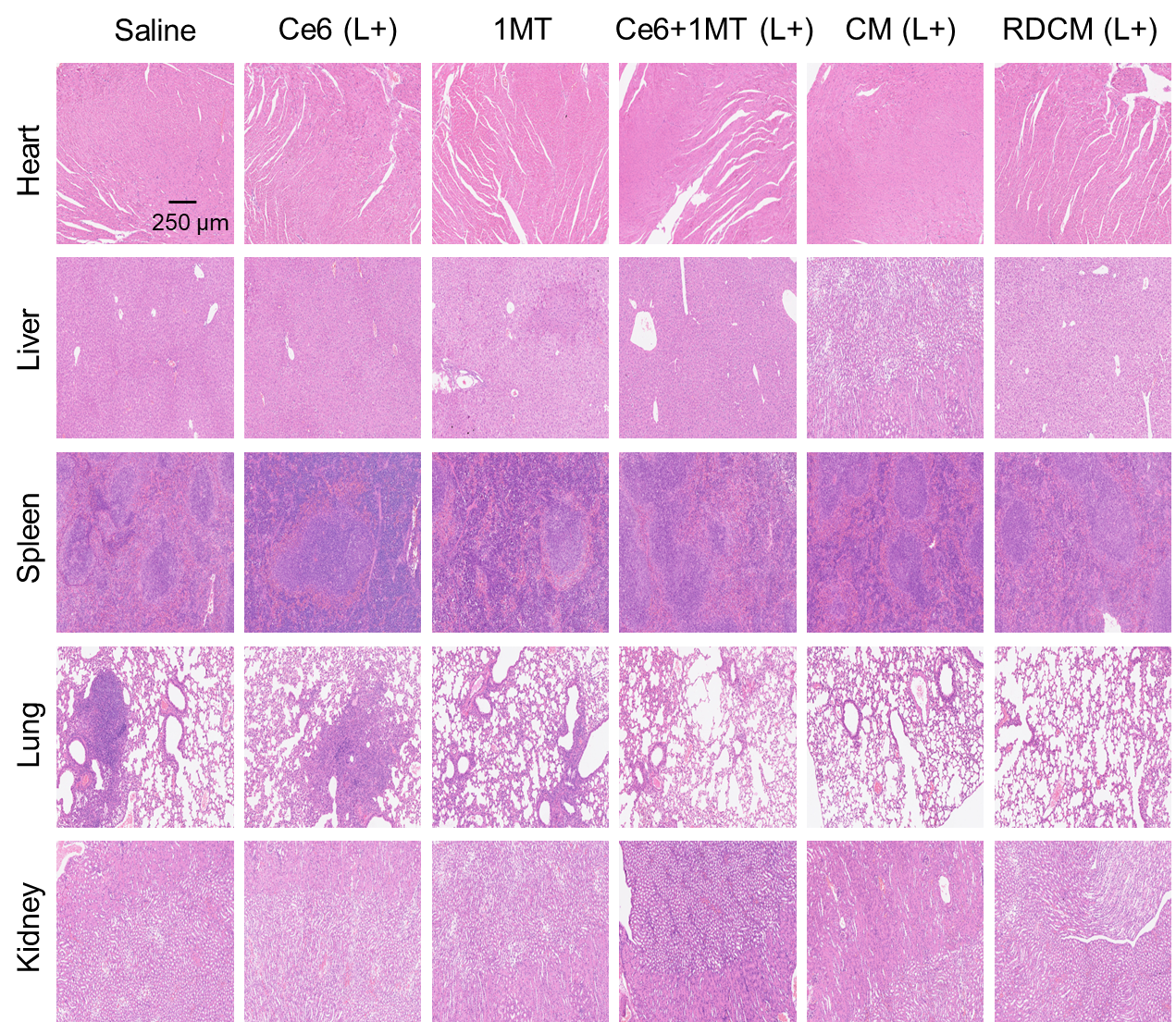


**Figure S7.** H&E staining images of major organs from mice at day 15.


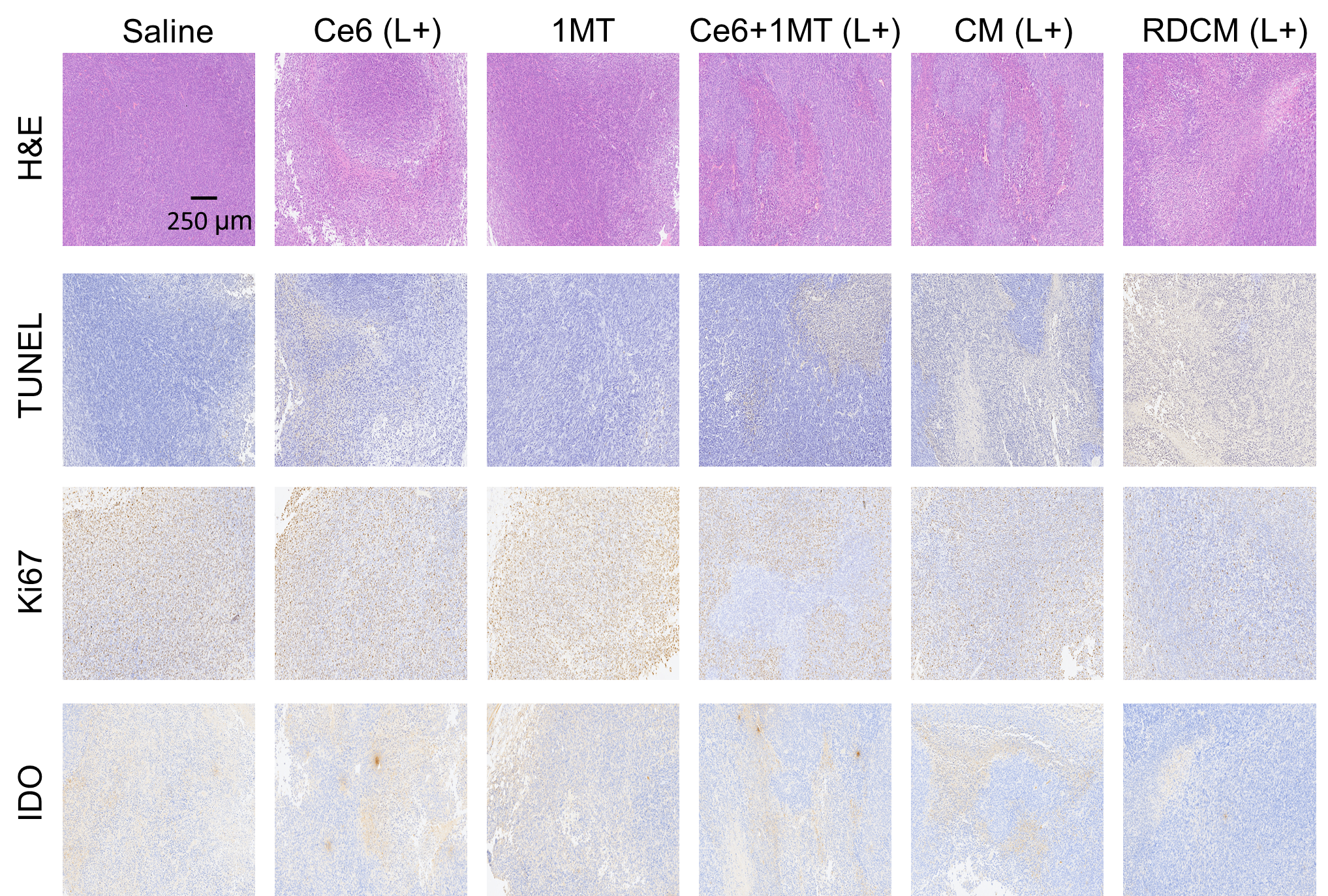


**Figure S8.** H&E staining, Ki67 analysis and TUNEL assay of the tumor slices of mice in different groups on day 15.

**
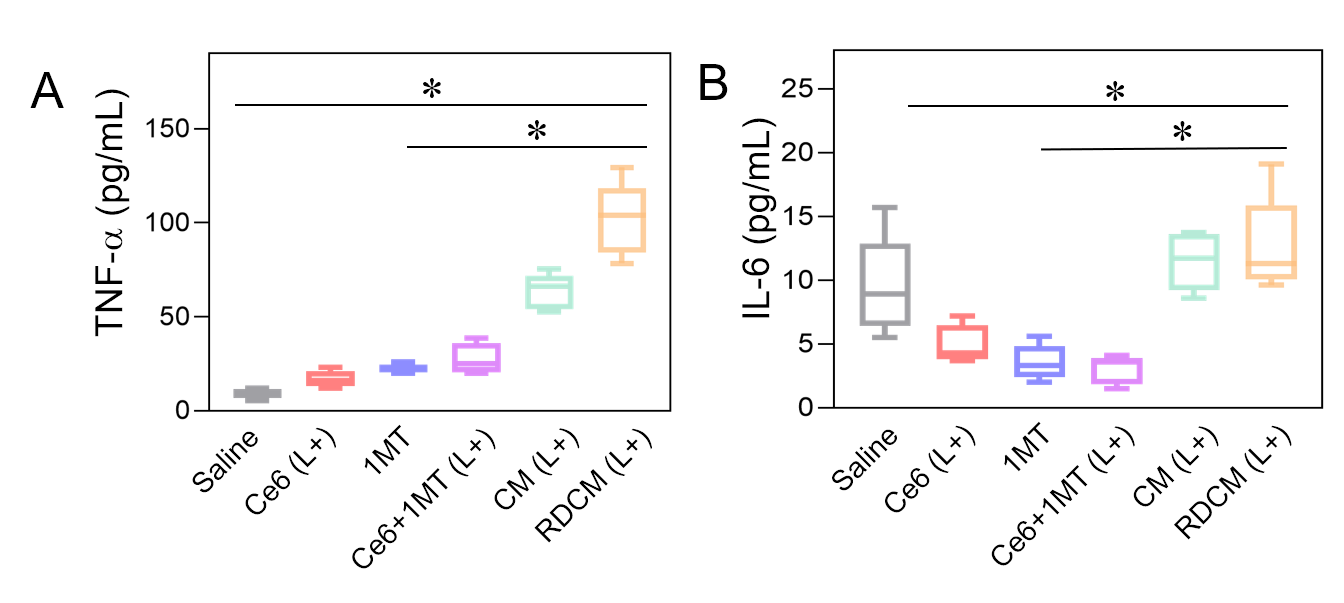
**

**Figure S9.** The ratio of Trp/Kyn (D), and the levels of TNF-α (E) and IL-6 (F) in the serum of mice (*n* = 4, **p*<0.05, ***p*<0.01).


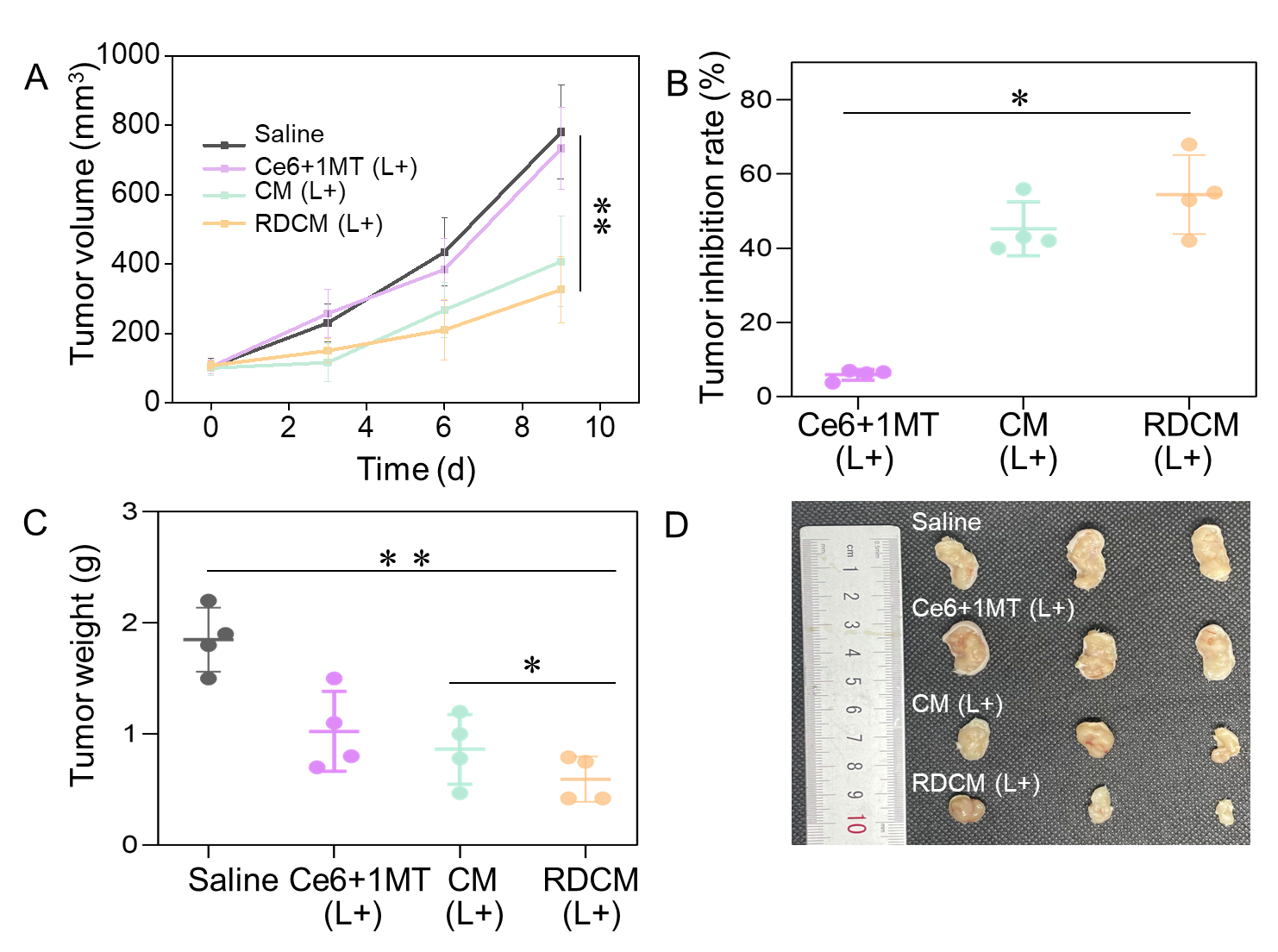


**Figure S10.** Tumor volumes (A), tumor inhibition rates (B), tumor weights (C) and photographs of representative tumors of mice in different groups (D), (*n* = 4, **p*<0.05, ***p*<0.01).


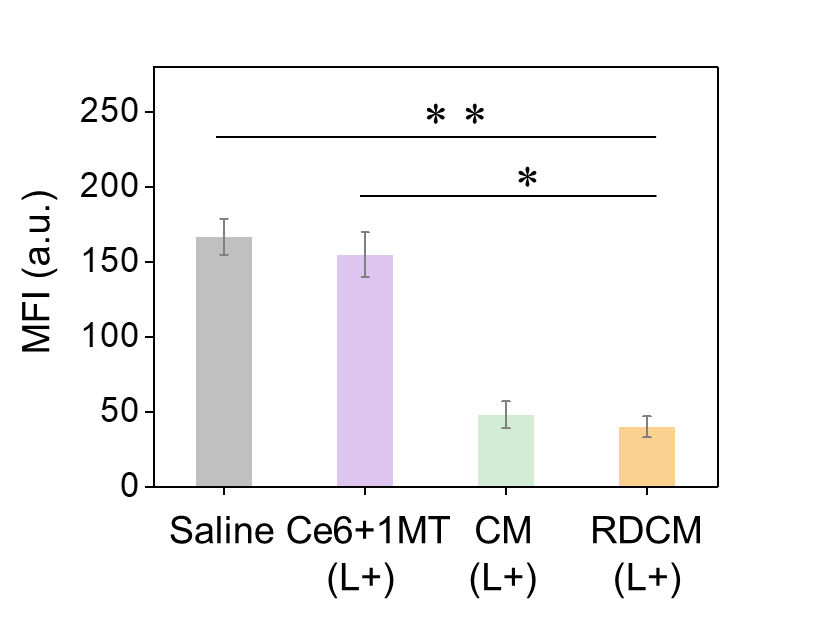


**Figure S11. S**emi-quantitative analysis of in vivo bioluminescence images of tumor-bearing mice at day 21.


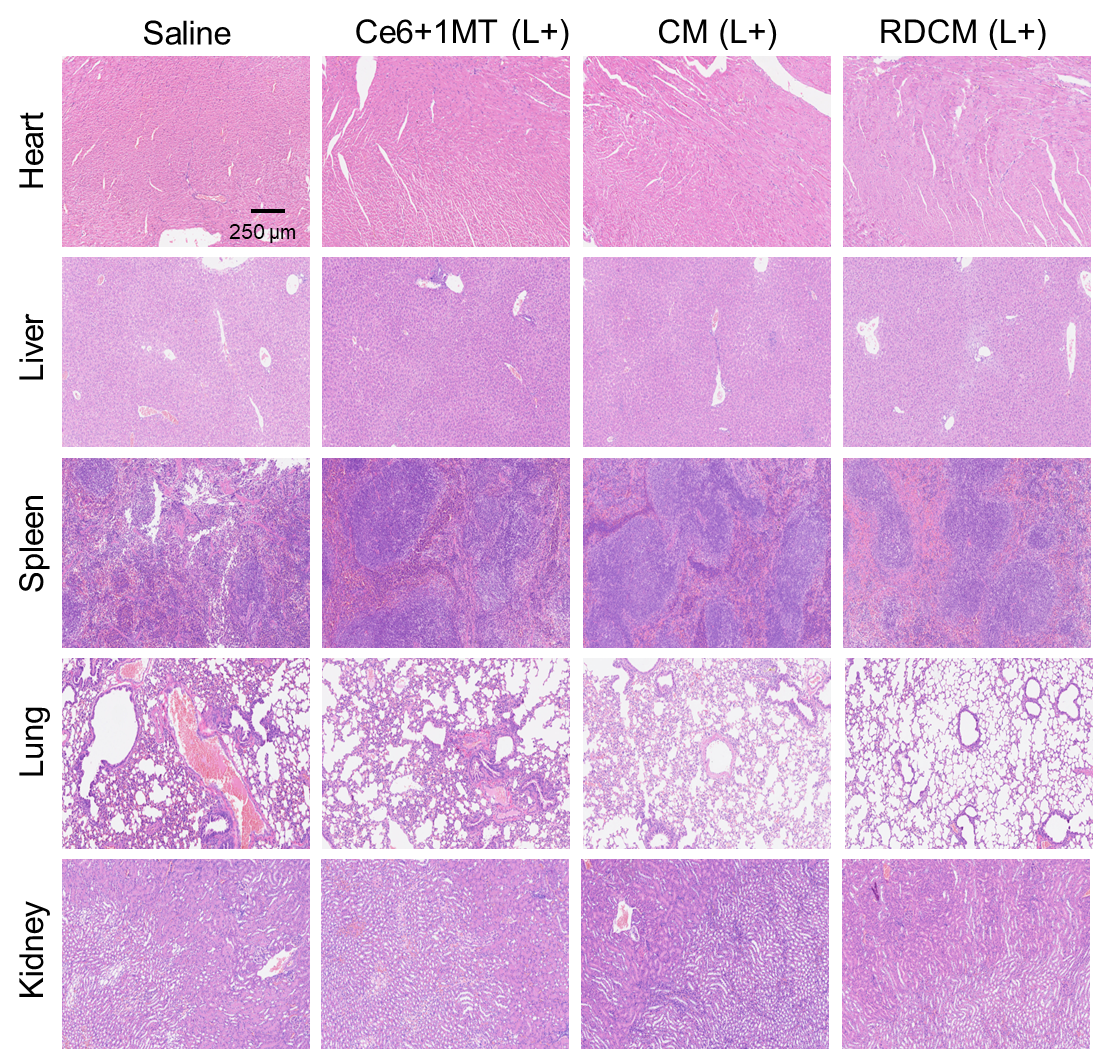


**Figure S12.** H&E staining images of major organs from tumor-bearing mice at day 27.**Table S1.** The drug loading contents (DLCs) of CM, RCM and RDCM.

| **Samples** | **CM** | | **RCM** | | **RDCM** | |
| --- | --- | --- | --- | --- | --- | --- |
| Feeding ratio of Ce6: 1MT^a^ | Ce6 (%) | 1MT (%) | Ce6 (%) | 1MT (%) | Ce6 (%) | 1MT (%) |
| 4:1 | 11.0 % | 2.3 % | 11.8 % | 1.7 % | 10.6 % | 2.0 % |
| 3:2 | 9.0 % | 5.3 % | 8.4 % | 3.6 % | 9.0% | 3.8 % |
| 2:3 | 5.7 % | 6.1 % | 6.0 % | 5.0 % | 5.7 % | 5.5 % |

a: The feeding weight ratios.
